# The FANCM Ortholog Fml1 Promotes Recombination at Stalled Replication Forks and Limits Crossing Over during DNA Double-Strand Break Repair

**DOI:** 10.1016/j.molcel.2008.08.024

**Published:** 2008-10-10

**Authors:** Weili Sun, Saikat Nandi, Fekret Osman, Jong Sook Ahn, Jovana Jakovleska, Alexander Lorenz, Matthew C. Whitby

**Affiliations:** 1 Department of Biochemistry, University of Oxford, South Parks Road, Oxford OX1 3QU, UK

**Keywords:** DNA

## Abstract

The Fanconi anemia (FA) core complex promotes the tolerance/repair of DNA damage at stalled replication forks by catalyzing the monoubiquitination of FANCD2 and FANCI. Intriguingly, the core complex component FANCM also catalyzes branch migration of model Holliday junctions and replication forks in vitro. Here we have characterized the ortholog of FANCM in fission yeast Fml1 in order to understand the physiological significance of this activity. We show that Fml1 has at least two roles in homologous recombination—it promotes Rad51-dependent gene conversion at stalled/blocked replication forks and limits crossing over during mitotic double-strand break repair. In vitro Fml1 catalyzes both replication fork reversal and D loop disruption, indicating possible mechanisms by which it can fulfill its pro- and antirecombinogenic roles.

## Introduction

Homologous recombination (HR) plays a central role in genome maintenance, being required for the faithful repair of DNA double-strand breaks (DSBs), as well as the processing of perturbed replication forks (McGlynn and Lloyd, 2002; San Filippo et al., 2008). The molecular details of HR are best understood in terms of DSB repair. Here resection of the broken DNA generates a single-stranded tail with a 3′-OH terminus. Rad51 then loads onto the tail to catalyze pairing and strand invasion with an intact homologous DNA molecule. Strand invasion forms a displacement loop (D loop) where DNA synthesis is primed from the 3′-OH end of the invading strand. At this point repair may diverge into a number of potential pathways, some of which result in conservation of the original parental DNA molecules (noncrossovers), whereas others lead to the reciprocal exchange of DNA flanking the break site (crossovers).

Specialized DNA helicases/translocases and endonucleases, which process D loops and their derivatives (Holliday junctions [HJs]), appear to function in competing pathways driving toward either noncrossover or crossover production. Crossovers are formed when D loops or HJs are cleaved by structure-specific endonucleases such as Mus81-Eme1 (Osman et al., 2003). This is important during meiosis for the formation of chiasmata that help direct correct homolog segregation. However, in mitotic cells crossing over is potentially hazardous if recombination occurs between the homologs or repeated DNA sequences, as this can give rise to loss of heterozygosity and gross chromosome rearrangement, respectively, which are events that are associated with disease such as cancer. It is probably for this reason that noncrossover pathways, such as synthesis-dependent strand annealing (SDSA) and double HJ dissolution, predominate in mitotic cells (Lorenz and Whitby, 2006).

Enzymes that process D loops/HJs can also play important roles in the processing of perturbed replication forks. For example, Mus81-Eme1 can cleave stalled replication forks to initiate repair by HR (Osman and Whitby, 2007). DNA helicases/translocases that branch migrate HJs may also catalyze a process called fork reversal/regression, which converts a stalled replication fork into a “chicken foot” structure. This may serve several purposes, including providing room for repair enzymes to access lesions that have blocked replication fork progression; facilitating lesion bypass by template switching; and promoting the recombination-dependent restart of replication (McGlynn and Lloyd, 2002).

In eukaryotes a select group of DNA helicases/translocases have been shown to catalyze HJ branch migration in vitro. These include a RecQ family DNA helicase, BLM; a core HR protein, Rad54; and a postreplication repair (PRR) protein, Rad5 (Blastyak et al., 2007; Bugreev et al., 2006; Karow et al., 2000). In addition to HJ branch migration, Rad54 has also been shown to dissociate D loops in vitro (Bugreev et al., 2007), Rad5 to catalyze fork reversal (Blastyak et al., 2007), and BLM to catalyze D loop dissociation, fork reversal, and double HJ dissolution (Bachrati et al., 2006; Ralf et al., 2006; Mankouri and Hickson, 2007). These in vitro activities are consistent with genetic data, which indicate roles in crossover avoidance for RecQ helicases (Ira et al., 2003), control of conversion tract length for Rad54 (Kim et al., 2002), and promotion of lesion bypass for Rad5 (Blastyak et al., 2007).

Recently, FANCM, which contains the characteristic motifs of a DEAH box helicase, has joined the ranks of eukaryotic enzymes that catalyze the ATP-dependent branch migration of HJs in vitro (Gari et al., 2008). It also branch migrates model fork substrates, suggesting that it might catalyze fork reversal as well (Gari et al., 2008). FANCM is one of 13 proteins in which defects have been linked to the rare human genetic disease Fanconi anemia (FA) (Wang, 2007). FA is associated with progressive bone marrow failure, a variety of developmental abnormalities, and a high incidence of cancer. Cells from FA patients exhibit hypersensitivity to DNA crosslinking agents such as mitomycin C and cisplatin, indicating a defect in DNA repair.

In vivo FANCM is known to be a component of the FA core complex that catalyzes the monoubiquitination of FANCD2 and FANCI, which is important for DNA repair (Wang, 2007). However, its ATP-dependent activities, while necessary for resistance to mitomycin C, are not required for FANCD2 or FANCI monoubiquitination (Xue et al., 2008). This suggests that FANCM's branch migration activity is required for additional aspects of DNA repair. One possibility is that FANCM might modulate the structure of stalled replication forks to facilitate repair (Gari et al., 2008). Alternatively, FANCM might participate in a Rad51-dependent process. However, to date there are limited genetic data to support this idea. Indeed in chicken DT40 cells the repair of DSBs by HR appears to be normal in a FANCM mutant (Mosedale et al., 2005). Similarly, Mph1, which is a relative of FANCM in *Saccharomyces cerevisiae* that exhibits a 3′–5′ helicase activity in vitro (Prakash et al., 2005), is not required for heteroallelic recombination (Schurer et al., 2004). However, the hypermutator phenotype of an *mph1* mutant, which is epistatic with *rad51*, indicates that it functions in an error-free pathway of DNA damage repair/tolerance that involves HR (Schurer et al., 2004).

Here we shed light on the potential function of FANCM in HR by characterizing its homolog in *Schizosaccharomyces pombe* Fml1. We show that, like FANCM, Fml1 is required for resistance to crosslinking agents and can catalyze the ATP-dependent branch migration of HJs in vitro. Moreover, we present genetic data showing that it promotes Rad51-dependent recombination at stalled replication forks and limits crossing over during mitotic DSB repair. Finally, we demonstrate that it catalyzes both fork reversal and D loop disruption in vitro and discuss how these activities can explain its pro- and antirecombinogenic activities.

## Results

### Identification of FANCM-like Proteins in *S. pombe*

**Figure 1:**
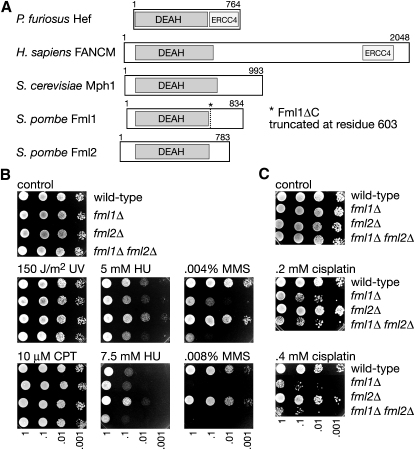
Identification of a FANCM Ortholog in *S. pombe* (A) FANCM orthologs. DEAH helicase and ERCC4 nuclease domains are indicated. Note that the ERCC4 nuclease domain in FANCM is thought to be inactive. (B and C) Spot assays. The strains are MCW1221, MCW2080, MCW2078 and MCW2082.

To identify potential orthologs of FANCM in *S. pombe*, we used BLAST to search the *S. pombe* gene database using the human FANCM amino acid sequence (accession no. AAZ53290) as bait. Two proteins, SPAC9.05 and SPAC20H4.04, were identified sharing 38% and 35% identity, respectively, with the helicase domain of FANCM (Figure 1A and S1 [available online]). We have named these *F*ANC*M*-*l*ike proteins Fml1 (SPAC9.05) and Fml2 (SPAC20H4.04). Both Fml1 and Fml2 also share significant homology between themselves and with other related DNA helicases, including *S. cerevisiae* Mph1 and *Pyrococcus furiosus* Hef (Figures 1A and S1).

To ascertain whether Fml1 or Fml2 is important for the repair/tolerance of DNA damage, we constructed *S. pombe* strains in which the *fml1* or *fml2* open reading frames were replaced with a selectable marker, and then tested these strains for their sensitivity to various genotoxins. Both mutant strains are viable and have no overt morphological or growth defects apart from a greater proportion of slightly elongated cells in the *fml1Δ* mutant (data not shown). Neither of the single mutants nor the *fml1Δ fml2Δ* double mutant exhibit hypersensitivity to ultraviolet light (UV), the topoisomerase 1 poison camptothecin (CPT), or the ribonucleotide reductase inhibitor hydroxyurea (HU) (Figure 1B). However, the *fml1Δ* mutant is hypersensitive to the alkylating agent methyl methanesulfonate (MMS) and the crosslinking agent cisplatin (Figures 1B and 1C). In contrast, the *fml2Δ* mutant is not hypersensitive to these agents, and in fact grows slightly better than wild-type in the presence of HU (Figures 1B and 1C). The reason for this improvement in growth in HU is currently unclear. The deletion of *fml2Δ* does slightly increase the sensitivity of a *fml1Δ* mutant to MMS, suggesting that there might be some functional redundancy between Fml1 and Fml2 (Figure 1B). However, overexpression of Fml2 does not suppress the MMS hypersensitivity of a *fml1Δ* mutant (data not shown), so if Fml2 can catalyze the same reaction as Fml1 then it is not simply a lack of expression that prevents it from substituting for Fml1 in vivo. Taken together, these data indicate that Fml1 plays a role in the repair/tolerance of specific types of DNA damage. As *FANCM* mutant cells exhibit hypersensitivity to crosslinking agents (Meetei et al., 2005), our data indicate that Fml1 is more likely to be a functional equivalent of FANCM than Fml2 in *S. pombe*, and therefore we decided to focus our analysis on this protein.

### Fml1 Promotes Gene Conversion at Blocked Replication Forks

**Figure 2:**
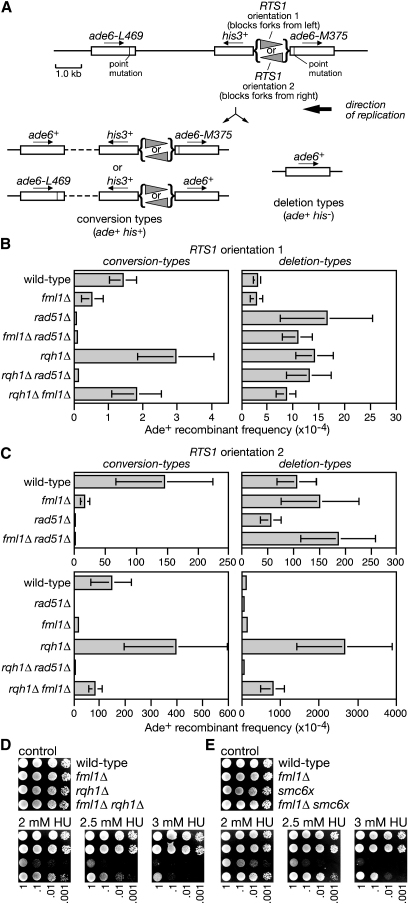
Fml1 Promotes Spontaneous and RFB-Induced Recombination (A) Schematic showing the recombination substrate on chromosome 3 plus the two types of recombinant product. (B and C) Ade^+^ recombinant frequencies for strains (B) MCW1262, MCW3059, MCW1691, MCW3790, MCW1443, MCW2132, and MCW3444, and (C) MCW1433, MCW3061, MCW1692, MCW3794, MCW1447, MCW2130, and MCW3456. Error bars are the standard deviations about the mean. (D and E) Spot assays of strains (D) MCW1193, MCW2096, MCW1818, and MCW2487, and (E) MCW1221, MCW2080, MCW1712, and MCW3701.

Both FANCM and Fml1 can branch migrate HJs in vitro, suggesting that they might be important in HR (Gari et al., 2008, and see later). Moreover, the hypersensitivity of the *fml1Δ* mutant to MMS and cisplatin suggests that Fml1 could play a role in processing blocked replication forks, because 3-methyladenines formed by MMS and crosslinks formed by cisplatin are potent fork barriers. To see whether Fml1 affects HR at blocked replication forks, we used strains in which a direct repeat of *ade6^−^* heteroalleles was integrated at the *ade6* locus on chromosome 3 with a polar replication fork barrier (RFB) *RTS1* positioned between them (Figure 2A) (Ahn et al., 2005). Due to the relative disposition of replication origins flanking *ade6*, this region of chromosome 3 is replicated unidirectionally, and therefore only one orientation of *RTS1* (orientation 2) blocks replication between the repeats. When this happens inter/intra sister chromatid recombination is strongly induced between the repeats, which can be monitored through the appearance of Ade^+^ prototrophs. Two classes of Ade^+^ recombinant can be distinguished by scoring the loss or retention of a *his3^+^* gene positioned between the repeats. Ade^+^ His^+^ recombinants (conversion types) are formed by Rad51-dependent gene conversion events, whereas Ade^+^ His^-^ recombinants (deletion types), occur by both Rad51-dependent and independent pathways (Ahn et al., 2005) (Figures 2B and 2C). In the absence of *fml1* the normal low frequency of spontaneous conversion types, which may arise from fork stalling at lesions that occur during normal growth, is significantly reduced by ∼3-fold compared to wild-type (Figure 2B and Table S1). However, this reduction is even greater (>8-fold relative to wild-type) when forks are blocked at *RTS1* (Figure 2C and Table S1). In contrast, there is no significant effect of *fml1Δ* on the frequency of deletion types when *RTS1* is in orientation 1, and only a small increase when it is in orientation 2 (Figures 2B and 2C and Table S1). Finally, we tested recombinant formation in a *fml1Δ rad51Δ* double mutant. As expected the double mutant exhibited the same very low-level of conversion-type formation as the *rad51Δ* single mutant for both spontaneous and *RTS1*-induced recombination, and essentially the same hyperlevel of spontaneous deletion types (Figures 2B and 2C and Table S1). However, surprisingly, the level of *RTS1*-induced deletion types was ∼3-fold more than that in the *rad51Δ* single mutant (Figure 2C and Table S1). Taken together, these data indicate that Fml1 plays an important role in promoting Rad51-dependent gene conversion at blocked replication forks. However, importantly, it also limits the formation of deletion types at blocked replication forks in the absence of Rad51, and the significance of this is discussed later.

### Fml1 Promotes Toxic Recombination at Stalled Replication Forks in *rqh1Δ* and *smc6x* Mutants

In *S. pombe* both the RecQ family DNA helicase Rqh1 and the structural maintenance chromosome protein Smc6 are important for limiting the accumulation of toxic Rad51-dependent recombination intermediates following replication fork stalling in HU (Laursen et al., 2003; Ampatzidou et al., 2006). Evidence for this comes in part from the observation that deletion of *rad51* suppresses the HU hypersensitivity of both *rqh1Δ* and *smc6x* mutants (Laursen et al., 2003; Ampatzidou et al., 2006). Similarly, deleting *fml1* partially suppresses the HU hypersensitivity of both *rqh1Δ* and *smc6x* mutants (Figures 2D and 2E). These data are consistent with Fml1 promoting Rad51-dependent recombination at stalled replication forks.

### Suppression of *rqh1Δ* Hyperrecombination

The deletion of *rqh1* results in elevated levels of both spontaneous and replication fork block-induced recombinant formation in the direct repeat assay (Figures 2B and 2C) (Ahn et al., 2005). We suspected that this hyperrecombination would be Rad51 dependent and therefore might also depend on Fml1. In order to test this, we first measured recombinant formation in the *rqh1Δ rad51Δ* double mutant (Figures 2B and 2C). As expected, conversion types depend on Rad51. Importantly, the very high levels of deletion types induced by replication fork blockage at *RTS1* are also Rad51 dependent (Figure 2C). In contrast, the spontaneous deletion types appear to be Rad51 independent; however, a requirement for Rad51 here may be masked by the fact that the *rad51Δ* mutant itself exhibits hyperlevels of deletion types (Figure 2B). Having established that the hyperrecombination in a *rqh1Δ* mutant is mostly Rad51 dependent, we next tested the *fml1Δ rqh1Δ* double mutant. Both spontaneous conversion types and deletion types are reduced by ∼1.5-fold in the double mutant relative to the *rqh1Δ* single mutant, and in both cases this reduction is statistically significant (p ≤ 0.001) (Figure 2B and Table S1). However, the reduction is even more striking for *RTS1* replication fork block-induced recombination, where conversion types are reduced by ∼5-fold and deletion types by >3-fold (Figure 2C). Altogether, these data indicate that Fml1 plays an important role in promoting the hyperlevels of Rad51-dependent recombination in a *rqh1Δ* mutant, particularly at blocked replication forks. Moreover, unlike in wild-type cells Fml1 promotes both conversion types and deletion types in a *rqh1Δ* mutant, and the potential significance of this is discussed later.

### Fml1 Limits Crossing Over during Mitotic DSB Repair

**Figure 3:**
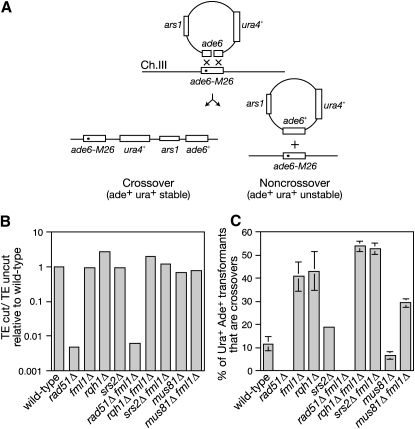
Fml1 Limits Crossing Over during Mitotic DSB Repair (A) Schematic showing the repair of a double-strand gap in *ade6* on plasmid pAN1 by homologous recombination with *ade6-M26* on chromosome 3. The *M26* mutation is indicated by the filled circle. See Figure S3 for a full description of the assay system. (B) Histogram showing the mean relative transformation efficiency (TE) of cut versus uncut plasmid in strains MCW1193, MCW2498, MCW2096, MCW1818, MCW3811, MCW2498, MCW2487, MCW2550, FO1192, and MCW2264. (C) Histogram showing the percentage of Ade^+^ recombinants that are crossovers in the same strains as in (B). Error bars are the standard deviations about the mean.

Having established that Fml1 promotes Rad51-dependent recombination at stalled replication forks, we next investigated whether it is required during DSB repair. For this we used a plasmid gap repair assay, which measures the repair of a linearized plasmid through recombination with homologous DNA on the chromosome (Orr-Weaver and Szostak, 1983) (Figure 3A). In our case the plasmid is cut within a copy of *ade6*, which has a 153 bp interstitial deletion at the cut site. The cut plasmid is then transformed into strains where it can be repaired via gene conversion with a mutant allele of *ade6* (*ade6-M26*) on chromosome 3, giving rise to Ade^+^ transformants. Typically between 70% and 80% of transformants are Ade^+^ in a HR-proficient strain, and this is true of all the strains that we are testing here except those containing a deletion of *rad51*, where no Ade^+^ transformants are recovered (Figure S2). To assess the efficiency of DSB repair, we performed parallel transformations with uncut plasmid to derive a ratio for the number of cut plasmid transformants to uncut plasmid transformants. This ratio was essentially the same in both wild-type and *fml1Δ* mutant, indicating that Fml1 is not essential for DSB repair (Figure 3B). In contrast, the cut-to-uncut plasmid transformant ratio is more than a 100-fold lower in a *rad51Δ* mutant, consistent with DSB repair depending on HR (Figure 3B).

Two types of Ade^+^ transformant can be distinguished in the plasmid gap repair assay—those where the plasmid has recircularized and is maintained autonomously (noncrossovers), and those where it has integrated into the chromosome (crossovers) (Figures 3A and S3). In wild-type cells only 10% of Ade^+^ recombinants are crossovers, whereas ∼40% are crossovers in a *fml1Δ* mutant, and as in the wild-type they are dependent on Rad51 (Figure 3C). These data indicate that Fml1 plays an important role in driving the processing of recombination intermediates into noncrossover products. In *S. cerevisiae* crossing over is limited during mitotic DSB repair by the Srs2 and Sgs1 DNA helicases (Ira et al., 2003). Similarly, both Srs2 and Rqh1 (an ortholog of Sgs1) limit crossing over during plasmid gap repair in *S. pombe*, albeit Srs2's role here is relatively minor compared to Rqh1 or Fml1 (Figure 3C). To see if Fml1 functions in a pathway of crossover avoidance that involves either Rqh1 or Srs2, we measured the percentage of Ade^+^ recombinants that are crossovers in both *fml1Δ rqh1Δ* and *fml1Δ srs2Δ* double mutants (Figure 3C). Both double mutants exhibit a significant (p ≤ 0.05) increase in crossovers relative to their respective single mutant strains, indicating that Fml1 functions in a pathway of crossover avoidance that is distinct, at least in part, from those involving Rqh1 and Srs2. Finally, we tested whether crossover formation depends on Mus81. As shown previously, Mus81 activity accounts for ∼50% of crossovers in wild-type cells (Figure 3C) (Osman et al., 2003). Similarly, the high level of crossing over in a *fml1Δ* mutant is partly Mus81 dependent, although it would seem that Mus81-independent crossover formation is prevalent.

### Fml1 Branch Migrates Synthetic Holliday Junctions In Vitro

**Figure 4:**
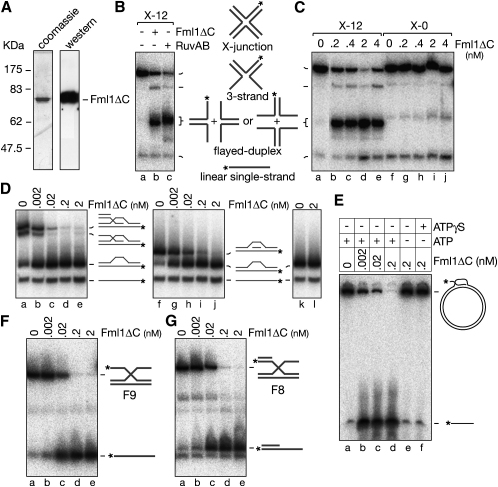
Catalysis of HJ Branch Migration and D Loop Dissociation by Fml1ΔC (A) Coomassie blue-stained SDS gel and immunoblot (probed with anti-polyhistidine) showing purified His-tagged Fml1ΔC. (B) PhosphorImage showing dissociation of X-12 by Fml1ΔC (2 nM) and RuvAB (40 nM RuvA and 630 nM RuvB). The schematic shows the various products of X-junction dissociation. Asterisks indicate ^32^P label at the DNA 5′ end. (C) Comparison of the dissociation of X-12 and X-0 by Fml1ΔC. (D) Dissociation of static D loops by Fml1ΔC. The substrates are D2 (lanes a–e), D7 (lanes f–j), and D8 (lanes k–l). The schematics show the DNA substrates and their various dissociation products, with the asterisks indicating the ^32^P label at the 5′ end of oligo 16. (E) Dissociation of a mobile D loop by Fml1ΔC. Reactions were incubated for 15 min at 37°C. (F and G) Dissociation of the part X-junctions F8 and F9 by Fml1ΔC.

It was shown recently that full-length FANCM can branch migrate HJs in vitro (Gari et al., 2008). In order to see whether Fml1 has the same ability, we attempted to express it with an N-terminal hexa-histidine tag in *Escherichia coli* with the view to purifying sufficient quantities for in vitro assays. However, despite trying several different *E. coli* strains designed to facilitate the production of full-length eukaryotic proteins, little or no soluble full-length protein was produced (data not shown). However, we were able to express and purify a C-terminally truncated form (Fml1ΔC-truncated at amino acid 603), which retains the conserved helicase domain (Figures 1A and 4A). Overexpression of Fml1ΔC fully complements the MMS hypersensitivity of a *fml1Δ* mutant, indicating that the critical biological function of Fml1 is retained (Figure S4).

Having confirmed that Fml1ΔC is active in vivo, we next tested whether the purified protein branch migrates HJs in vitro. For this we used the synthetic HJ X-12, which contains a central 12 bp core of homology, in which the junction point can branch migrate, flanked by 18 bp of heterology in each junction arm. HJ branch migration enzymes like *E. coli* RuvAB characteristically dissociate X-12 into flayed duplexes by branch migrating the junction point and unwinding of a pair of heterologous arms in a reaction that depends on ATP hydrolysis. Like RuvAB, Fml1ΔC can perform this reaction (Figure 4B, compare lanes b and c), and as expected it depends on Mg^2+^ and a hydrolysable form of ATP (Figure S5A). Having established that Fml1ΔC can catalyze HJ branch migration, we next tested whether it could dissociate X-0, which shares sequence similarity with X-12 but lacks the core of homology, meaning that its junction point is fixed (Figure 4C). Unlike X-12, which is readily dissociated by substoichiometric amounts of Fml1ΔC (lanes b–e), X-0 is not unwound (lanes g–j), even though both junctions are bound equally well, as judged by an electrophoretic mobility shift assay (EMSA) (Figure S5B). These data indicate that Fml1ΔC has a limited processivity for DNA unwinding. This is reminiscent of FANCM, which has little or no DNA unwinding activity and therefore is only capable of dissociating junctions with almost perfect homology in their arms (Gari et al., 2008).

### Fml1ΔC Dissociates D Loops In Vitro

Having established that Fml1ΔC can branch migrate HJs, we next tested its activity on D loops. First, we used a static D loop (D2) made from oligonucleotides, where each section of duplex DNA is 20 bp. Fml1ΔC readily dissociates this substrate by unwinding the invading strand (Figure 4D, lanes b–e). It also unwinds an invading strand with no exposed single-stranded or double-stranded arm (lanes g–j), but not the duplex regions flanking the D loop (lane l). We next tested Fml1ΔC on a mobile D loop made by RecA-catalyzed strand invasion of a radiolabeled 100-mer oligonucleotide into a supercoiled plasmid DNA (Figure 4E). Again Fml1ΔC readily dissociates this substrate (lanes b–d) in a reaction that is dependent on ATP (lane e) and cannot be substituted by its poorly hydrolysable analog ATPγS, indicating that ATP hydrolysis is required (lane f). Finally, we determined whether Fml1ΔC dissociates D loops by unwinding the invading strand from its 3′ end or from its four-way junction end. For this we constructed a series of static substrates that resemble the junctions at either end of a D loop. Substrates F8 and F9 resemble the four-way junction end of the D loop, consisting of an invading strand with a duplex or single-stranded arm, respectively (Figure 4F and G). In both cases Fml1ΔC specifically unwinds the equivalent of the invading strand (Figures 4F and 4G, lanes b–e). In contrast, Fml1ΔC is unable to unwind a static junction that resembles the 3′ invading end of a D loop, at least in a way that is consistent with D loop dissociation (Figures 6B, 6F, and 6G). Altogether these data indicate that Fml1ΔC can utilize its ability to branch migrate/unwind four-way DNA junctions to efficiently dissociate D loops in vitro.

### Fml1ΔC Promotes Fork Reversal In Vitro

An ability to dissociate D loops might explain how Fml1 limits crossing over during DSB repair. However, it is not consistent with Fml1's prorecombinogenic role at stalled replication forks. It would seem that here Fml1 must have additional or alternative functions. It was recently suggested that FANCM might aid the repair of lesions that cause replication fork blockage by catalyzing fork reversal (Gari et al., 2008). Fork reversal is a potential mechanism by which HR can be stimulated—it generates a double-stranded DNA end, which after appropriate processing can form a 3′-ended single-stranded tail onto which Rad51 can load and promote strand invasion (McGlynn and Lloyd, 2002). If Fml1 can catalyze fork reversal, then this might explain how it can promote the formation of conversion-type recombinants in our direct repeat assay (see Figure S6).

**Figure 5:**
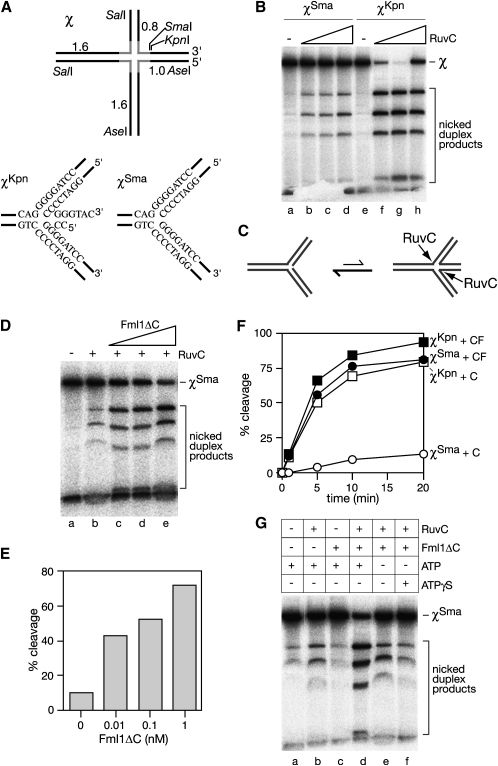
Fml1ΔC Catalyzes Fork Reversal (A) Schematic of χ-DNA showing the position of restriction sites used to generate χ^Kpn^ and χ^Sma^. The homologous core (gray lines) and size (in kb) of each duplex arm are indicated. (B) Comparison of χ^Sma^ and χ^Kpn^ cleavage by RuvC. (C) Schematic illustrating equilibrium between forms of χ^Sma^ that are cleavable and non-cleavable by RuvC. (D) Stimulation of RuvC cleavage of χ^Sma^ by increasing amounts of Fml1ΔC (0.01, 0.1 and 1 nM). (E) Quantification of the data in (D). (F) Rates of χ^Sma^ and χ^Kpn^ cleavage by RuvC in the presence and absence of Fml1ΔC. “C” and “F” denote RuvC and Fml1ΔC respectively. Values are means of two experiments (error bars are omitted for clarity). (G) Dependence on ATP hydrolysis for Fml1ΔC-stimulated cleavage of χ^Sma^ by RuvC.

To see whether Fml1 has the ability to catalyze fork reversal, we used an in vitro assay that was originally developed for the analysis of the RecG DNA helicase from *E. coli* (McGlynn and Lloyd, 2000). This uses χ-DNA, which contains a HJ within a central core of 312 bp of homology flanked by heterologous duplex arms of ∼0.65–1.45 kb, each with a ^32^P label at its terminus (Figure 5A). Digestion of this χ-DNA with KpnI (to generate χ^Kpn^) cleaves off most of one arm but leaves a small amount of heterology that prevents branch migration of the HJ into a pseudo replication fork (Figure 5A). In contrast, cleavage with SmaI (to generate χ^Sma^) removes the same junction arm precisely at the boundary of the homology, enabling a fork substrate to be generated by branch migration (Figure 5A). As shown previously, χ^Sma^ is cleaved poorly by the *E. coli* HJ resolvase RuvC, whereas χ^Kpn^ is cleaved comparatively well (Figure 5B, compare lanes b–d with f–h) (McGlynn and Lloyd, 2000). Poor cleavage of χ^Sma^ is explained by it preferentially adopting the pseudo replication fork structure, which RuvC is unable to cleave (Figure 5C). However, the addition of an enzyme that catalyzes fork reversal, like RecG, converts the fork into a HJ and thereby stimulates cleavage of χ^Sma^ (McGlynn and Lloyd, 2000). Similar to RecG, increasing amounts of Fml1ΔC stimulate χ^Sma^ cleavage by RuvC (Figure 5D, lanes c–e, and Figure 5E). In fact, the addition of Fml1ΔC stimulates the rate of cleavage of χ^Sma^ by >10-fold, making it comparable to the rate of cleavage of χ^Kpn^ (Figure 5F).

To confirm that Fml1ΔC stimulates RuvC cleavage of χ^Sma^ by actively catalyzing fork reversal rather than by modulating the fork through DNA binding, we tested whether the reaction depends on ATP hydrolysis. As shown in Figure 5G, there is no stimulation of RuvC cleavage if ATP is either removed or replaced with the poorly hydrolysable analog ATPγS (compare lanes d–f). In fact in the presence of ATPγS Fml1ΔC inhibits the already low level of χ^Sma^ cleavage by RuvC. This inhibition is also seen with χ^Kpn^ (Figure S7) and is probably due to Fml1ΔC more stably binding to junction DNA in the presence of ATPγS. This could inhibit cleavage either by blocking RuvC's access to the DNA or limiting junction branch migration to nucleotide sequences that RuvC can cut. The data in Figure 5G also confirm that the increase in χ^Sma^ cleavage is not due to any nuclease activity associated with our purified Fml1ΔC (compare lanes a and c). Altogether, these data show that Fml1ΔC can catalyze fork reversal in vitro and therefore may do the same in vivo.

### Fml1ΔC Can Unwind the Lagging Strand at a Fork Structure

**Figure 6:**
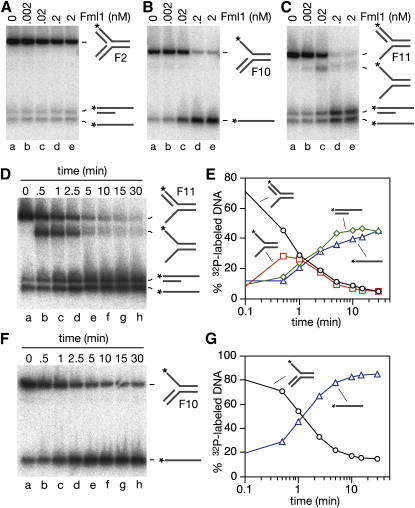
Lagging Strand Unwinding Catalyzed by Fml1ΔC (A–C) Unwinding assays showing Fml1ΔC's ability to dissociate fork substrates F2, F10 and F11. Schematics of the substrates and reaction products are shown with asterisks indicating the ^32^P label at the DNA 5′ end. (D and F) Time courses of F11 and F10 unwinding. Reactions (100 μl) contain Fml1ΔC (2 nM) and ^32^P labeled F11 or F10 (0.5 nM) as indicated. (E) Quantification of data in (D). (G) Quantification of data in (F).

The fork structure in χ^Sma^ has no single-stranded DNA exposed at the junction point, and as such may resemble certain types of blocked replication fork in vivo. However, for replication forks blocked by lesions in the leading template strand, polymerase uncoupling may generate forks where the 5′ end of the nascent lagging strand is positioned ahead of the blocked leading strand (Pages and Fuchs, 2003). To see if Fml1ΔC can unwind a fork structure like this, we used substrates made from similar oligonucleotides as those in X-0. Fml1ΔC therefore has to unwind 24–25 bp in any junction arm to generate a dissociation product. The first fork (F2) contains both leading and lagging nascent strand mimics, and even though Fml1ΔC binds this substrate (Figure S8), it is completely resistant to dissociation (Figure 6A). In contrast, when the lagging strand is removed to make junction F10, Fml1ΔC is able to unwind the fork (Figure 6B, lanes b–e). However, instead of unwinding the leading strand it unwinds the equivalent of the template strands. It also does this to the fork without the leading strand (junction F11), but here the detection of flayed duplex DNA indicates that it unwinds the lagging strand as well (Figure 6C, lanes b–e). In fact, a time course of the dissociation of this fork shows that the lagging strand is unwound at least 4× faster than the duplex in front of the fork (Figure 6D, lanes b–h, and Figure 6E). It is also faster than the dissociation of junction F10 (Figures 6F and 6G). Figure 6E also shows that both the flayed duplex produced by unwinding the lagging strand and the partial linear duplex that is generated from unwinding the template strands in front of the fork are themselves unwound in secondary reactions to generate single strands (note that the flayed duplex products that are generated from the dissociation of X-12 [Figures 4B and 4C] are not unwound in a secondary reaction because their duplex regions exceed the limit of Fml1's DNA-unwinding activity, which is between 25 and 30 bp [data not shown]). Overall, these data establish that Fml1 would be capable of unwinding at least a limited stretch of nascent lagging strand at a stalled replication fork. However, the ability of Fml1 to dissociate F11 in either of two ways indicates that its initial orientation of binding is not dictated by the asymmetry of the junction, which suggests that additional factors might be needed in vivo to ensure that Fml1 binds in a way that is productive for promoting fork reversal.

### Genetic Interaction with Other Junction-Processing Enzymes

**Figure 7:**
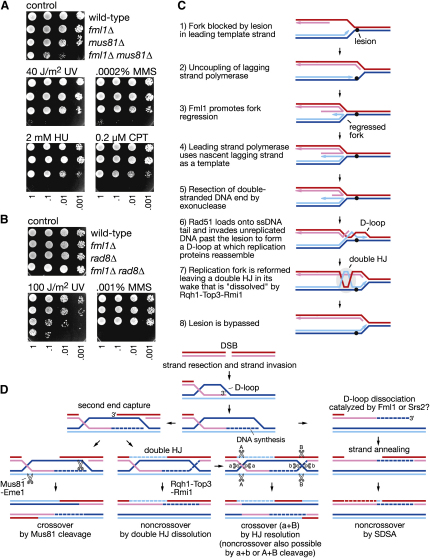
Genetic Interactions with Other Junction Processing Enzymes and Models for Fml1's Roles in Replication Fork Processing and DSB Repair (A) Spot assay of strains MCW1221, MCW2080, MCW1238, and MCW2428. (B) Spot assay of strains MCW1221, MCW2080, MCW3781, and MCW3816. (C) Model for lesion bypass promoted by Fml1. (D) Model showing different pathways of DSB repair.

If Fml1 processes forks and/or D loops in vivo, then its function might overlap with other proteins that are known to process these substrates. Any overlap should be detectable by a more than additive (i.e., synergistic) increase in genotoxin sensitivity when the single gene deletions are combined. Based on this analysis, we have already shown that Fml1's function does not overlap with that of Rqh1 with respect to the response to HU (Figure 2D), and the same is true for UV, MMS, and CPT (data not shown). Three additional proteins were tested: Rad54, whose human ortholog can branch migrate HJs and dissociate D loops in vitro (Bugreev et al., 2007; Bugreev et al., 2006); Mus81, which can cleave forks, D loops, and HJs (Osman and Whitby, 2007); and Rad8, which is the ortholog of *S. cerevisiae* Rad5 that catalyzes fork reversal and promotes an error-free branch of PRR (Blastyak et al., 2007). In the case of Rad54, no reduction in viability or additional sensitivity to UV, MMS, HU, or CPT was observed in the *fml1Δ rad54Δ* double mutant relative to a *rad54Δ* single mutant (data not shown). In contrast, the *fml1Δ mus81Δ* double mutant is poorly viable and is considerably more sensitive to genotoxins than either *fml1Δ* or *mus81Δ* single mutants (Figure 7A). Likewise, the *fml1Δ rad8Δ* double mutant exhibits an increase in MMS and UV sensitivity relative to its single mutants (Figure 7B), although neither single nor double mutants exhibit hypersensitivity to HU or CPT (data not shown). These data are consistent with the idea that Fml1 promotes an alternative way of processing stalled forks and/or D loops to both Mus81-Eme1 and Rad8.

## Discussion

In an effort to understand the function of FANCM in humans, we have characterized its relative in *S. pombe* Fml1. As mentioned earlier, FANCM is a component of the so-called FA core complex, which plays an important role in the monoubiquitination of FANCD2 and FANCI (Wang, 2007). Apart from FANCM, *S. pombe* does not have obvious homologs of FANC proteins, and therefore its use as a model organism to study all aspects of the FA pathway is clearly limited. Nevertheless, recent data indicate that the ATP-dependent activities of FANCM, while necessary for resistance to the crosslinking agent mitomycin C, are not required for monoubiquitination of FANCD2 and FANCI (Xue et al., 2008). This suggests that FANCM might perform other important roles in DNA repair, and it is these additional functions that we think are conserved in *S. pombe*. In support of this view we have shown that Fml1, like FANCM, is required for resistance to crosslinking agents such as cisplatin and can catalyze the ATP-dependent branch migration of HJs in vitro. We therefore believe that our data, which show that Fml1 promotes Rad51-dependent recombination at stalled/blocked replication forks and limits crossing over during DSB repair, are relevant to understanding the role(s) of FANCM in humans.

### Promoting Rad51-Dependent Recombination at Stalled/Blocked Replication Forks by Catalyzing Fork Reversal

One of the important findings of our study is that Fml1 promotes the formation of both the low level of conversion-type recombinants that occur spontaneously during normal growth and the very high level that occur following replication fork blockage at *RTS1*. The formation of conversion types depends on strand invasion catalyzed by Rad51 (Ahn et al., 2005; Doe et al., 2004), and therefore Fml1 is in some way promoting this activity. One possibility is that Fml1 acts as a general accessory factor for HR, aiding processes like Rad51 nucleofilament assembly, strand invasion, or strand exchange. However, loss of proteins that perform these functions, such as Rad55, Rad57, and Rad54, results not only in a reduction in conversion-type formation, but also a marked increase in spontaneous deletion types similar to a *rad51Δ* mutant (Doe et al., 2004). The deletion types stem from single-strand annealing (SSA), which increases when Rad51 activity is impaired. The fact that *fml1* deletion fails to cause an increase in spontaneous deletion types indicates that Rad51 is not generally dependent on Fml1 for its activity. A view supported by our observation that Fml1 is not needed for *RTS1*-induced Rad51-dependent deletion-type formation, nor for efficient plasmid gap repair, which depends on Rad51. It also concurs with genetic studies in DT40 cells and *S. cerevisiae* that show no dependence on FANCM/Mph1 for the recombinational repair of DSBs (Mosedale et al., 2005; Schurer et al., 2004).

Fml1's ability to branch migrate/unwind HJs and forks suggests at least two non-mutually exclusive ways in which it might promote Rad51-dependent conversion types at stalled/blocked replication forks: (1) it could promote strand exchange by its HJ branch migration activity; and (2) it could generate a substrate for HR by catalyzing fork reversal. In contrast, D loop dissociation would appear to be inconsistent with a role in promoting gene conversion, which depends on strand invasion. Indeed, a reduction in D loop dissociation would be expected to result in an increase in recombination as observed in cells lacking Srs2, which is thought to disrupt D loops (Doe and Whitby, 2004; Dupaigne et al., 2008). Moreover, Fml1's shared ability with Rad51 to promote HU hypersensitivity in *rqh1Δ* and *smc6x* mutants suggests that it promotes the formation of toxic recombination intermediates, rather than disrupts them. It would seem therefore that Fml1's ability to promote recombination at stalled replication forks overrides any antirecombinogenic ability it might have.

We believe that fork reversal can best account for Fml1's prorecombinogenic activity, as this explains how it can promote Rad51-dependent gene conversion at stalled replication forks without acting as a general accessory factor for HR. Here Fml1 provides the substrate (an exposed double-stranded DNA end) for recombination rather than directly driving Rad51 activity (Figure S6). Evidence that Fml1 might act directly on stalled replication forks comes from our observation that deletion of *fml1* results in an increase in replication fork block-induced deletion types in the absence of Rad51. This shows that Fml1 can function independently of Rad51 at blocked replication forks. We suspect that Rad51 loads at blocked forks regardless of whether Fml1 has promoted their reversal. Loading of Rad51 without fork reversal could help protect the fork, as has been suggested for RecA in *E. coli* (Courcelle and Hanawalt, 2003), but may be suboptimal for promoting the formation of conversion types. In the absence of Rad51, fork reversal by Fml1 may help to protect the fork from cleavage by endonucleases like Mus81-Eme1 that preferentially act on forks rather than HJs (Osman and Whitby, 2007). In this way deletion-type formation by SSA may be averted.

As noted above, Fml1 is generally not needed for deletion-type formation following replication fork blockage at *RTS1*. One exception is in a *rqh1Δ* mutant. Here, the high-levels of deletion types may arise, at least in part, from a failure of Rqh1 to process Rad51-dependent strand invasion events that stem directly from Fml1-promoted fork reversal. Cleavage of these recombination intermediates by Mus81-Eme1 could account for the deletion types (Figure S6). Unfortunately, the inviability of a *rqh1Δ mus81Δ* double mutant prevents us from directly testing this idea.

### The Purpose of Fork Reversal In Vivo

As discussed above, Fml1 promotes Rad51 activity at stalled replication forks, and this can be explained by it catalyzing fork reversal. We believe that the purpose of this reaction is to promote the repair and/or tolerance of certain types of DNA lesion (e.g., 3-methyladenines induced by MMS) that block replication fork progression. Tolerance could be achieved by fork reversal, enabling polymerases to bypass damaged sections of the template by switching to the equivalent undamaged nascent strand (Figure 7C) (Higgins et al., 1976). Following template switching (step 4), the reversed fork would have to be reset and replication restarted. This could occur by Rad51-mediated strand invasion of the template DNA ahead of the lesion, which would generate a D loop at which the replisome could reassemble (step 6) (McGlynn and Lloyd, 2002). Replication restart by this method would result in a double HJ behind the replication fork, which could be removed by the Rqh1-Top3-Rmi1 “dissolvase” (steps 7–8) (Mankouri and Hickson, 2007).

We believe that the failure of this pathway can account for the MMS hypersensitivity of a *fml1Δ* mutant and the synergistic increase in MMS sensitivity of a *fml1Δ rad8Δ* double mutant. In the latter case we assume that Rad8 catalyzes fork reversal like its ortholog in *S. cerevisiae* Rad5, which does so to promote HR-independent error-free PRR by a template switching mechanism similar to that proposed here for Fml1 (Blastyak et al., 2007). Inappropriate use of the above pathway can also explain the suppression of *rqh1Δ* and *smc6x* HU sensitivity by *fml1Δ*. Here fork reversal results in pathological structures when the mechanisms of fork stabilization and/or recombination intermediate processing are impaired. A failure of fork reversal may also account for the hypermutator phenotype of *fml1Δ* and the *S. cerevisiae mph1* mutant, where there is an increase in translesion DNA synthesis when template switching is impaired (Schurer et al., 2004; W.S. and M.C.W., unpublished data). We also suspect that a similar pathway is promoted by FANCM in humans, and in this regard it is interesting to note that the FA core complex copurifies with the Bloom syndrome complex, which contains the orthologs of Rqh1 (=BLM), Top3 (=Top3α), and Rmi1 (=BLAP75) (Wang, 2007). However, it does not readily account for FANCM's requirement for interstrand crosslink repair. Here fork reversal may promote repair by providing room for repair enzymes to access the lesion (Gari et al., 2008), as well as a substrate for Rad51 to promote replication restart.

### D Loop Dissociation and Crossover Avoidance

In addition to promoting Rad51-dependent gene conversion at stalled replication forks, Fml1 also plays a major role in limiting crossing over during mitotic DSB repair. Importantly, the pathway of crossover avoidance that is promoted by Fml1 is, at least in part, independent from those promoted by Rqh1 and Srs2. Rqh1, like other RecQ helicases, probably limits crossing over by catalyzing double HJ dissolution (Figure 7D). In contrast, Srs2 is thought to limit crossing over by promoting SDSA, and one way that it might do this is by dissociating D loops (Figure 7D). Fml1's ability to dissociate D loops in vitro suggests that it too might limit crossing over by promoting SDSA (Figure 7D). If correct then, our genetic data indicate that Fml1 is the major mechanism for driving SDSA in *S. pombe*. In its absence we suspect that the processing of D loops becomes more reliant on Mus81-Eme1, which would explain the poor viability and extreme genotoxin sensitivity of the *fml1Δ mus81Δ* double mutant. However, deletion of *mus81* only reduces crossover formation by less than half in a *fml1Δ* mutant, indicating that crossovers can be made independently of Mus81-Eme1.

Currently it is not known whether FANCM can dissociate D loops in vitro, but conservation of HJ branch migration between Fml1 and FANCM suggests that it might. If this proves to be the case, then FANCM, like Fml1, might promote SDSA, which might in turn explain the elevated levels of spontaneous sister chromatid exchange in FANCM-deficient DT40 cells (Mosedale et al., 2005).

### Conclusion

We have shown that Fml1 has both pro- and antirecombinogenic activities, which are likely to result from its ability to reverse replication forks and dissociate D loops, respectively. The concept of Fml1 using its branch migration/helicase activity in such distinct ways is not without precedence. Indeed, it is reminiscent of *E. coli* RecG, which harnesses its branch migration activity to both reverse forks and dissociate R loops (McGlynn and Lloyd, 2000; Vincent et al., 1996). How Fml1's opposing activities in HR promotion and control are appropriately coordinated, particularly in relationship to other junction-processing enzymes, is an important question that requires further investigation. Finally, we suspect that Fml1's dual function in both promoting and controlling HR is conserved in FANCM—highlighting potentially important ways in which it might maintain genome stability beyond simply stimulating the monoubiquitination of FANCD2 and FANCI.

## Experimental Procedures

The genetic methods, strains, plasmids, spot assays, purification of proteins, and DNA substrates are described in the Supplemental Experimental Procedures.

### Recombination Assays

Direct repeat recombination was assayed by measuring the frequency of Ade^+^ recombinants as described (Ahn et al., 2005). Recombinant frequencies represent the mean value from at least 15 colonies for each strain. The plasmid gap repair assay was based on the assay developed by Orr-Weaver and Szostak (1983), and is described in the Supplemental Experimental Procedures. For both assays, two sample t tests were used to determine the statistical significance of differences in recombinant frequencies between strains.

### Branch Migration/Unwinding Assays

Reaction mixtures (20 μl) contained 0.5 nM labeled substrate DNA (or 40 pM of plasmid-based D loop) in binding buffer (50 mM Tris-HCl [pH 8.0], 1 mM DTT, 100 μg/ml BSA, 6% glycerol) plus 2.5 mM MgCl_2_, 5 mM ATP, and protein as indicated. The RuvAB reaction in Figure 4B contains 5 mM MgCl_2_ and 1 mM ATP. Reactions were incubated at 30°C for 30 min (unless otherwise stated) and then terminated by adding one-fifth volume of stop mix (2.5% SDS, 200 mM EDTA, 10 mg/ml proteinase K) and incubating for a further 15 min at 37°C to deproteinize the mixture. Products were analyzed by electrophoresis through 6% or 10% native polyacrylamide gels at 200 V in TBE buffer. Gels were dried on 3 MM Whatman paper and analyzed with a Fuji FLA3000 PhosphorImager.

### χ Cleavage Assays

Reaction mixtures (20 μl) contained ^32^P-labeled χ DNA in binding buffer plus 5 mM MgCl_2_, 5 mM ATP, and protein as indicated. Reactions were typically preincubated with Fml1ΔC for 5 min at 37°C prior to the addition of 0.1 nM RuvC and continued incubation at 37°C for 30 min. Reactions were terminated as described above and analyzed by electrophoresis through 0.8% agarose gels in TBE buffer at 100 V for 80 min. Gels were dried and analyzed as above.

## Supplemental Data

Document S1. Supplemental Experimental Procedures, Eight Figures, and Three Tables
