# Supplementary material for: The FANCM Ortholog Fml1 Promotes Recombination at Stalled Replication Forks and Limits Crossing Over during DNA Double-Strand Break Repair
Source: Mol Cell. 2008 Oct 10;32(1-24):118–28. doi: 10.1016/j.molcel.2008.08.024 (PMC2581491; doi:10.1016/j.molcel.2008.08.024)
Supplement: Document S1. Supplemental Experimental Procedures, Eight Figures, and Three Tables [file mmc1.pdf]

Molecular Cell, Volume *32*

Supplemental Data

The FANCM Ortholog Fml1 Promotes Recombination at Stalled Replication Forks and Limits Crossing Over during DNA Double-Strand Break Repair

Weili Sun, Saikat Nandi, Fekret Osman, Jong Sook Ahn, Jovana Jakovleska, Alexander Lorenz, and Matthew C. Whitby

SUPPLEMENTAL EXPERIMENTAL PROCEDURES

Media and genetic methods

Media and genetic methods used standard protocols (Moreno et al., 1991). The complete and minimal media were yeast extract with supplements (YES) and Edinburgh minimal medium plus 3.7 mg/ml sodium glutamate (EMMG) plus appropriate amino acids (0.2475 mg/ml), respectively. Low adenine media (YELA) was supplemented with 0.01 mg/ml adenine. Ade^+ recombinants were selected on YES lacking adenine and supplemented with 200 mg/l guanine to prevent uptake of residual adenine.

Strains and plasmids

Strains are listed in Table S2. The *fml1Δ::natMX4* and *fml2Δ::kanMX6* strains were made by gene targeting using derivatives of pAG25 and pFA6-KanMX6, respectively (Bahler et al., 1998; Goldstein and McCusker, 1999). The full-length *fml1* cDNA was cloned by reverse transcription PCR, using primers oMW1035 (5' – TATTACATATGTCCGATGATTCTTTTAG – 3') and oMW1036 (5' – TATTGGATCCATTCTCTAAATCAGCATTCC – 3'), into pET14b (Novagen) to make pSN2, which expresses Fml1 with an N-terminal hexa-histidine tag from the T7 phage Ø10 promoter. pMW855 was made by subcloning *fml1* from pSN2 into pREP41. *fml1ΔC* was constructed from pSN2 using primers oMW1035 and oMW1060 (5' – GGGATCCCTAATTTTCTTTGGGAATTTC – 3') and cloned into pET14b and pREP41 to make the Fml1ΔC expression plasmids pSN3 and pMW856, respectively. pAN1 is a derivative of pREP42 containing a copy of *ade6* (with a deletion of a 153 bp internal *Nco*I fragment) cloned between the *Pst*I and *Sac*I sites.

Spot assays

Exponentially growing cells from liquid cultures were harvested, washed and resuspended in water at a density of $1 \times 10^7 – 1 \times 10^3$ cells/ml. Aliquots (10 µl) of the cell suspensions were spotted onto agar plates containing genotoxins as indicated. For UV, plates were irradiated using a Stratalinker (Stratagene). Plates were photographed after 3 - 6 days growth at 30˚C.

Plasmid gap repair assay

Logarithmically growing strains were transformed with either 4 μg of *Nco*I-linearized pAN1 or uncut pAN1 essentially as described by Keeney and Boeke (1994) except that carrier DNA was omitted. Cells were plated onto EMMG plates lacking uracil (EMMG-u) and incubated at 30˚C for 5 - 8 days. The numbers of transformants were then counted to determine the relative transformation efficiency of cut versus uncut plasmid. This provides a measure of how well the cut plasmid is repaired. The cut plasmid transformants were then patched onto EMMG-u, incubated for 3 days at 30˚C, and replica plated onto EMMG lacking both adenine and uracil (EMMG-u-a) to score the number of Ade^{+} recombinants amongst the Ura^{+} transformants. The patched transformants on EMMG-u were also replica plated onto YELA plates, which after 3 days at 30˚C were re-replica plated onto YELA. Following these two rounds of growth on non-selective media the YELA patches were replica plated onto complete media containing 5'-fluoroorotic acid (FOA), which counter-selects Ura^{+} cells. Growth on FOA indicates that *ura4*$^{+}$ has not integrated into the chromosome (noncrossover) and therefore is readily lost without selection, whereas poor growth on FOA indicates that *ura4*$^{+}$ is not easily lost due to its integration into the chromosome via crossover recombination. Assays were repeated at least three times for each strain. In each assay 300 Ura^{+} transformants were patched and assessed for Ade^{+} and crossover status by the replica plating protocol described above.

Proteins

RuvA and RuvB were a gift from Robert Lloyd (University of Nottingham), and RecA was from New England Biolabs. The purification of Fml1ΔC and RuvC are described below. Amounts of protein are expressed in moles of monomer, and were estimated using a Bio-Rad protein assay kit with bovine serum albumin as the standard.

Purification of RuvC

RuvC was overexpressed from plasmid pGS775 in BL21 (DE3) pLysS as described (Dunderdale et al., 1994). Cell lysis, and the precipitation of RuvC and its subsequent re-dissolving were all essentially as described (Dunderdale et al., 1994). However, the re-dissolved RuvC was loaded onto a 5 ml HiTrap Blue column (Pharmacia Biotech) pre-equilibrated in R buffer (20 mM Tris-HCl, pH 8.0, 1 mM EDTA, 1 mM dithiothreitol (DTT) and 10% (v/v) glycerol) containing 0.5 M KCl. RuvC eluted from this column between 0.6 – 0.7 M KCl, and was then dialysed against R buffer containing 0.3 M KCl before loading onto a 1 ml HiTrap Heparin column (Pharmacia Biotech). The peak of RuvC (1.5 ml) eluted from this column at ~750 mM KCl. This sample was mixed with 1.5 ml of 100% (v/v) glycerol, and stored as aliquots at –80˚C.

Purification of FmlΔC

2-litre cultures of *E. coli* Rosetta(DE3)pLysS (Novagen) containing pSN3 were grown with aeration at 30 °C in Luria-Bertani broth containing 125 μg/ml ampicillin and 34 μg/ml chloramphenicol. At a cell density corresponding to an A_{600} of 0.6, Fml1ΔC was induced by adding isopropyl-1-thio-β-D-galactopyranoside to a final concentration of 0.5 mM, following which the cells were incubated for a further 7 hrs. The cells were then harvested by centrifugation, resuspended in Buffer H (50 mM potassium phosphate, pH 8.0, 0.3 M NaCl, 10% glycerol), and frozen at -80 °C until required. All of the subsequent steps were at 4 °C. The defrosted cells were mixed with 1% Triton X-100, 10

mM β-mercaptoethanol and protease inhibitors before passage through a French pressure cell at 30,000 p.s.i.. Cell debris was then removed by centrifugation at 43,700 x *g* for 50 min, and the supernatant was loaded directly onto a 2 ml nickel-nitrilotriacetic acid (Ni-NTA) Superflow column (Qiagen) that was washed with 60 ml of Buffer H plus 20 mM imidazole before eluting bound Fml1ΔC with Buffer H plus 200 mM imidazole into 2 ml fractions. The second 2 ml fraction contained the peak of Fml1ΔC and was loaded directly onto a HiLoad 16/60 Superdex 200 gel filtration column (Amersham Biosciences), which was then developed with 120 ml of Buffer A (50 mM Tris-HCl, pH8.0, 1 mM EDTA, 1 mM DTT, 10% glycerol) plus 0.3 M NaCl. 2 ml fractions were collected and the peak of Fml1ΔC eluted between fractions 33 - 38. These fractions were pooled, diluted with an equal volume of Buffer A, and loaded onto a 1 ml Hi-Trap Heparin column (GE Healthcare). The column was then washed with 5 ml of Buffer A plus 0.1 M NaCl before eluting bound protein with an 18 ml gradient from 0.1 to 1.0 M NaCl. The peak of Fml1ΔC eluted between 0.41 – 0.44 M NaCl, and these fractions were pooled and stored as aliquots at -80 °C.

DNA substrates

Oligonucleotides 1 - 8, 10, 15 - 19 and 22, used to make the X-junctions, part X-junctions fork substrates, and static D-loops are listed in Table S3. These oligonucleotides, together with the procedures for substrate preparation, have been described previously (Doe et al., 2002; Osman et al., 2003; Whitby and Dixon, 1998). The X-junctions, part X-junctions and fork substrates are ^{32}P-labelled at the 5′ end of oligonucleotide 2. The static D-loops are ^{32}P-labelled at the 5' end of oligonucleotide 16. Protocols for the construction of plasmid-based D-loops have been described previously (McIlwraith et al., 2001). In brief, 5' ^{32}P-labelled oligonucleotide oMW592 (5' – TGCCGAATTCTACCAGTGCACGCCTCCATCCAGTCTATTAATTGTTGCCGGGA AGCTAGAGTAAGTAGTTCGCCAGTTAATAGTTTGCGCAACGTTGTTG – 3') was incubated with RecA for 5 minutes at 37˚C before the addition of supercoiled pBR322 plasmid to initiate strand invasion. After 5 minutes the reaction was terminated by the addition of stop mix and incubating for a further 30 min at 37 °C to deproteinize the mixture. To purify D-loops the deproteinized reaction mixture was applied to a 3.5 ml sepharose CL-2B column, which was developed with 20 mM Tris-HCl (pH 8.0) and 0.5 mM $MgCl_2$. Substrate concentrations were determined with reference to the specific activity of the radiolabelled oligonucleotide. χ, χ^{Kpn} and χ^{Sma} substrates were made as described (McGlynn and Lloyd, 2000).

EMSA

Reaction mixtures (20 μl) contained 0.5 nM labeled substrate DNA in Binding Buffer (50 mM Tris-HCl, pH 8.0, 1 mM DTT, 100 μg/ml BSA, 6% glycerol). Reactions were started typically by the addition of Fml1ΔC, held on ice for 15 min, and then loaded immediately onto a pre-equilibrated 4% native polyacrylamide gel in low ionic strength buffer (6.7 mM Tris-HCl, pH 8.0, 3.3 mM sodium acetate, 2 mM EDTA). Samples were run into the gel typically for 1hr and 30 mins at 160 V with buffer recirculation occurring throughout. Both buffer and gel were pre-cooled at 4°C, but electrophoresis was at room temperature. Gels were dried on 3 MM Whatman paper, and analysed with a Fuji FLA3000 PhosphorImager.

SUPPLEMENTAL REFERENCES

Bahler, J., Wu, J. Q., Longtine, M. S., Shah, N. G., McKenzie, A., 3rd, Steever, A. B., Wach, A., Philippsen, P., and Pringle, J. R. (1998). Heterologous modules for efficient and versatile PCR-based gene targeting in *Schizosaccharomyces pombe*. Yeast *14*, 943-951.

Doe, C. L., Ahn, J. S., Dixon, J., and Whitby, M. C. (2002). Mus81-Eme1 and Rqh1 involvement in processing stalled and collapsed replication forks. J. Biol. Chem. *277*, 32753-32759.

Dunderdale, H. J., Sharples, G. J., Lloyd, R. G., and West, S. C. (1994). Cloning, overexpression, purification, and characterization of the *Escherichia coli* RuvC Holliday junction resolvase. J. Biol. Chem. *269*, 5187-5194.

Goldstein, A. L., and McCusker, J. H. (1999). Three new dominant drug resistance cassettes for gene disruption in *Saccharomyces cerevisiae*. Yeast *15*, 1541-1553.

Keeney, J. B., and Boeke, J. D. (1994). Efficient targeted integration at *leu1-32* and *ura4-294* in *Schizosaccharomyces pombe*. Genetics *136*, 849-856.

McIlwraith, M. J., Hall, D. R., Stasiak, A. Z., Stasiak, A., Wigley, D. B., and West, S. C. (2001). RadA protein from *Archaeoglobus fulgidus* forms rings, nucleoprotein filaments and catalyses homologous recombination. Nucleic Acids Res. *29*, 4509-4517.

Moreno, S., Klar, A., and Nurse, P. (1991). Molecular genetic analysis of fission yeast *Schizosaccharomyces pombe*. Methods Enzymol. *194*, 795-823.

Whitby, M. C., and Dixon, J. (1998). Substrate specificity of the SpCCE1 Holliday junction resolvase of *Schizosaccharomyces pombe*. J. Biol. Chem. *273*, 35063-35073.

```
Fml1     1 ---MSDDSFSSDE-----------DWDELDTQVVDKIENEYHNNTIGLNGYSVDEYFD--
Mph1     1 --MASADDYFSDF-----------EDDELDKLYEKAINKSVKETITRRAVPVQKDLHDNV
Fml2     1 MIVLRDSSDYSDSEVDLPSIEVNGERGNVPTSVLCNVEGSLLNSNPALRQPNLKCIKHNQ
FANCM    1 -MSGRQRTLFQTWG----------SSISRSSGTPGCSSGTERPQSPGSSKAPLPAAAEAQ

Fml1    45 --------------ANDSNRYRLQHELD--------ESAAQQWVYPINVSFRDYQFNIVQ
Mph1    48 LPGQKTVYEEI---QRDVSFGPTHHELD--------YDALSFYVYPTNYEVRDYQYTIVH
Fml2    61 DVENTTFQLDLRGCRVPSSQPQVITELENNIDIPKNIDAMQNWIFPQTQQYRNYQKEFCE
FANCM   50 LESDD----DVLLVAAYEAERQLCLENGG-----FCTSAGALWIYPTNCPVRDYQLHISR

Fml1    83 KALFENVLVALPTGLGKTFIAAVVMMNYLRWFPKSYIVFMAPTKPLVTQQMEACYKITGI
Mph1    97 KSLFQNTLCAIPTGMGKTFIASTVMLNYFRWTKKAKIIFTAPTRPLVAQQIKACLGITGI
Fml2   121 QALFHNLLLALPTGLGKTFIAAVVMLNYFRWFPESKIIFLAPTKPLLLQQRVACSNVAGM
FANCM  101 AALFCNTLVCLPTGLGKTFIAAVVMYNFYRWFPSGKVVFMAPTKPLVTQQIEACYQVMGI

Fml1   143 PKSQTAELSGHVPVTTRNQYYQSRNVFFVTPQTILNDIKHGICDRTRISCLVIDEAHRST
Mph1   157 PSDQTAILLDKS-RKNREEIWANKRVFFATPQVVENDLKRGVLDPKDIVCLVIDEAHRAT
Fml2   181 SPGATAELNGEVSPDRRLFEYNTKRVFFMTPQTLQNDLKEHLLDAKSIICLIFDEAHRAT
FANCM  161 PQSHMAEMTGSTQASTRKEIWCSKRVLFLTPQVMVNDLSRGACPAAEIKCLVIDEAHKAL

Fml1   203 GNYAYVEVVHLLSLSNKNFRILALSATPGNKLEAIQNVIDSLHISRIEIRTENSIDISQY
Mph1   216 GSSAYTNVVKFIDRFNSSYRLLALTATPASDLEGVQEVVNNLDISKIEIRTEESMDIVKY
Fml2   241 GNHSYAQVMRAVLRSNSHFRVLGLTATPGSSTASVQKVVDCLHISKLIVRNEESIDIRSY
FANCM  221 GNYAYCQVVRELVKYTNHFRILALSATPGSDIKAVQQVITNLLIGQIELRSEDSPDILTY

Fml1   263 VQKKEVDFFPVDLSAEITDIRDRFSSILEPMLQKLN-KGNYYRIQNAKDITSFTVVQAKQ
Mph1   276 MKKRKKEKIEVPLLLEIEDIIEQLGMAVKPVLQQAI-ELGIYEECDPSQINAFKAMQQSQ
Fml2   301 VFHKKIQLIKVTISSEMNILKSDFANLYRPYFNFLRQKKLIPINCECLNIKAYTLFVSLR
FANCM  281 SHERKVEKLIVPLGEELAAIQKTYIQILESFARSLI-QRNVLMRRDIPNLTKYQIILARD

Fml1   322 AFLAMSGQNFPANQKWDILNTFDALATFAYPLNLLLNHGIRPFYQKLREVEEECFVGRSG
Mph1   335 KIIAN--PTIPEGIKWRNFFILQLLNNVGQMLKRLKIYGIRTFFNYFQNKCTEFTTKYNL
Fml2   361 KYSFSS-KNVQSKEKSKIMSCFTLLISCAHITYLLDCHGIIQFYQKLVETKNKAEGKGSG
FANCM  340 QFRKNPSPNIVGIQQGIIEGEFAICISLYHGYELLQQMGMRSLYFFLCGIMDGTKGMTRS

Fml1   382 YKKR-----------------IINHENYRPLMDDIEILLRDQSFVG-HPKLEHLERIVTE
Mph1   393 KKSTNKI-----------AAEFYYHPILKNIKNQCENYLSDPKFVG-HGKLQCVRDELMD
Fml2   420 QSFW-----------------LFTSKPFAFYLEHLHNKIQGLSLN--HPKMNHLLELLKE
FANCM  400 KNELGRNEDFMKLYNHLECMFARTRSTSANGISAIQQGDKNKKFVYSHPKLKKLEEVVIE

Fml1   424 YFEKE--------QTKDTRIMIFVEIRSSAEEILRFLGKFYPN-VRPAIFIGQSAVRK--
Mph1   441 FFQK---------RGSDSRVIIFTELRESALEIVKFIDSVADDQIRPHIFIGQARAKEGF
Fml2   461 HFKDT------SEGYQNQRVMIFTEFRNTAEYITTTLLAIRPM-VRASLFIGQANSAYS-
FANCM  460 HFKSWNAENTTEKKRDETRVMIFSSFRDSVQEIAEMLSQHQPI-IRVMTFVGHASGKS--

Fml1   473 --------------------------------------------------------AAGM
Mph1   492 DEVKYTRKHAPKGRKKVERLHRQEQEKFLEAERTKRAANDKLERSARRTGSSEEAQISGM
Fml2   513 ---------------------------------------------------------TGM
FANCM  517 --------------------------------------------------------TKGF

Fml1   477 SQKLQNETVKQFQKGEVNTLIATSIGEEGLDIGEVDMIICYDASASPIRMLQRMGRTGRK
Mph1   552 NQKMQKEVIHNFKKGEYNVLVCTSIGEEGLDIGEVDLIICYDTTSSPIKNIQRMGRTGRK
Fml2   516 NQMQQKETIDQFRAGVINTLVATSIGEEGLDIGDTDMIICYDASSSPIRTIQRMGRTGRK
FANCM  521 TQKEQLEVVKQFRDGGYNTLVSTCVGEEGLDIGEVDLIICFDSQKSPIRLVQRMGRTGRK

Fml1   537 RKGYIYMLLTRGKEEAKWERAKDAYRTLQDNIVSG-RGLSLSEKSYRILPEKFRPVCDKR
Mph1   612 RDGKIVLLFS-SNESYKFERAMEDYSTLQALIS---KQCIDYKKSDRIIPEDIIPECHET
Fml2   576 KSGKVFVLLTEDCEDSKWERSQVSYRRVQKVIESG-KKIALKKDVPRLIPSNIQPIFKFQ
FANCM  581 RQGRIVIILSEGREERIYNQSQSNKRSIYKAISSNRQVLHFYQRSPRMVPDGINPKLHKM

Fml1   596 VIEIPKEN----------E-----E--VVV------------------APKKVQLRTKIK
Mph1   668 LITINDENEIINEMEDVDEVIRYATQCMMGKK---------VKPKKAITKKKRVQENKKP
Fml2   635 ALQNNADA------------------------------------------TLILNSYNNN
FANCM  641 FITHGVYEPEKPSRNLQRKSSIFSYRDGMRQSSLKKDWFLSEEEFKLWNRLYRLRDSDEI
```

Fig. S1. Sequence alignment of the helicase domains of Fml1, Fml2, Mph1 and human FANCM.

The alignment was constructed using ClustalW. Solid blocks indicate identical residues and grey blocks indicate similar residues.

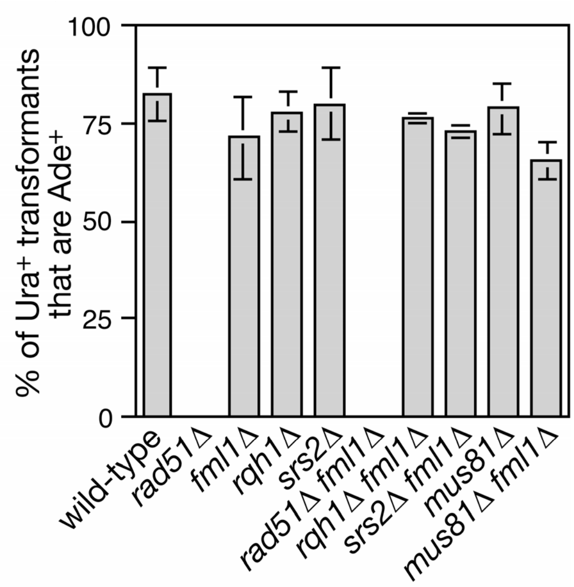

Fig. S2. Percentage of Ade^+ recombinants amongst Ura^+ transformants in the plasmid gap repair assay. The strains are: MCW1193, MCW2498, MCW2096, MCW1818, MCW3811, MCW2498, MCW2487, MCW2550, FO1192 and MCW2264. Error bars are the standard deviations about the mean.

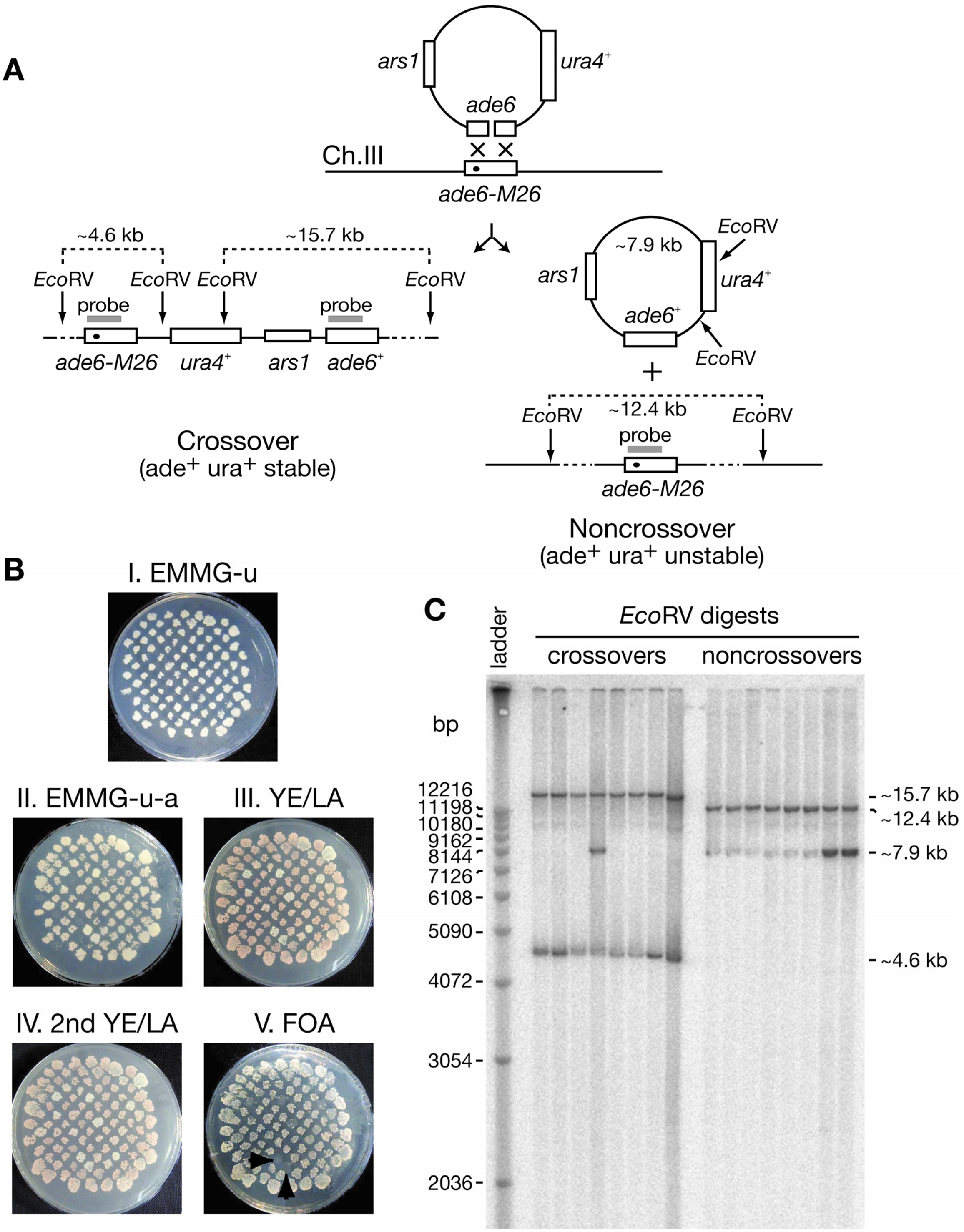

Fig. S3. Plasmid gap repair assay.

(A) Schematic showing the repair of a double-strand gap in *ade6* on plasmid pAN1 by homologous recombination with *ade6-M26* on chromosome 3. Repair of *ade6* can occur with or without crossing over. Crossing over results in the integration of the plasmid into the chromosome and therefore stability of the Ade$^+$ Ura$^+$ plasmid markers, whereas for noncrossovers the plasmid is maintained extrachromosomally due to the autonomously

replicating sequence *ars1*, and therefore can be lost if selection for it is not maintained (unstable Ade$^+$ Ura$^+$). The position of the *M26* mutation is indicated by the filled circle and is outside the position of the double-strand gap. Restriction sites, DNA probes, and fragment sizes, relevant to the southern blot analysis in (C), are indicated.

(B) An example of the analysis of plasmid gap repair assay transformants. (I) Ura$^+$ transformants are first patched onto minimal media lacking uracil (EMMG-u). (II) The number of ura$^+$ transformants that are ade$^+$ is assessed by replica plating the EMMG-u plate onto minimal media lacking uracil and adenine (EMMG-u-a). (III and IV) Two rounds of replica plating onto non-selective media (YELA) are performed to allow loss of non-integrated plasmid DNA. (V) The second YELA plate is replica plated onto FOA to identify stable and unstable transformants. Stable transformants remain ura$^+$ and therefore are unable to grow on FOA (examples indicated by black arrowheads), whereas, unstable transformants lose the *ura4*$^+$ gene and are therefore able to grow on FOA.

(C) Southern blot analysis of eight crossover transformants and eight noncrossover transformants identified by the protocol described in (B). Genomic DNA from eight stable Ura$^+$ Ade$^+$ and eight unstable Ura$^+$ Ade$^+$ transformants was digested with *Eco*RV, run on a agarose gel, southern blotted and probed with a fragment of *ade6* DNA as indicated in (A). In all cases the pattern of bands confirms the designation of transformants as crossover or noncrossover by the protocol in (B).

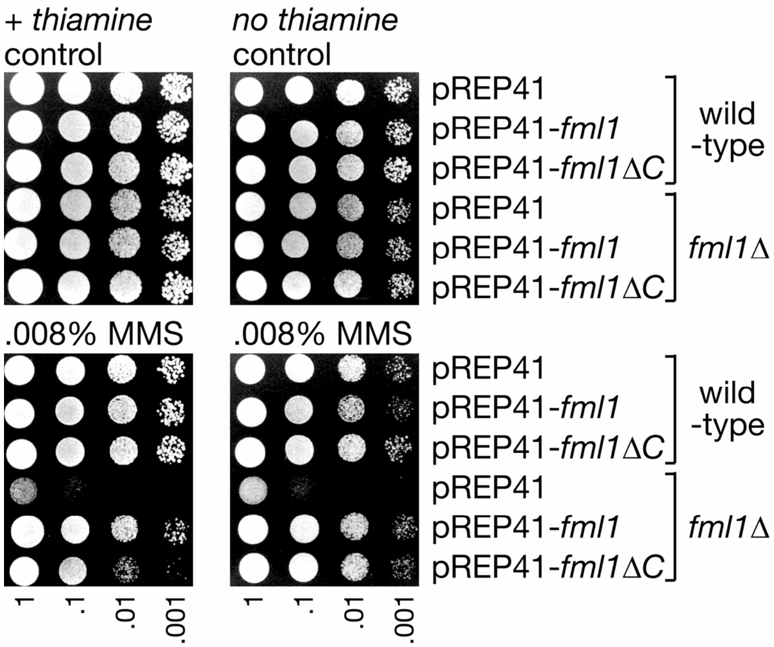

Fig. S4. Spot assay showing complementation of *fml1*Δ MMS sensitivity by full-length and truncated versions of Fml1. The strains are MCW1221 (wild-type) and MCW2080 (*fml1*Δ) transformed with plasmids as indicated.

To see whether Fml1ΔC retains activity *in vivo* we compared full-length and truncated Fml1 for their ability to complement the MMS hypersensitivity of the *fml1*Δ mutant. The proteins were expressed from the thiamine-repressible *nmt* promoter in pREP41, and in the absence of thiamine both fully complemented the *fml1*Δ mutant. Even in the presence of thiamine, the low-level of Fml1 that leaks from the repressed *nmt* promoter is sufficient to fully complement *fml1*Δ. In contrast, the repressed level of FmlΔC only partially complements. These data indicate that Fml1ΔC retains its core biological function, albeit it needs to be over expressed to be fully effective *in vivo*. Possibly the non-conserved C-terminal domain, which is deleted in Fml1ΔC, promotes protein stability or efficient targeting of substrates.

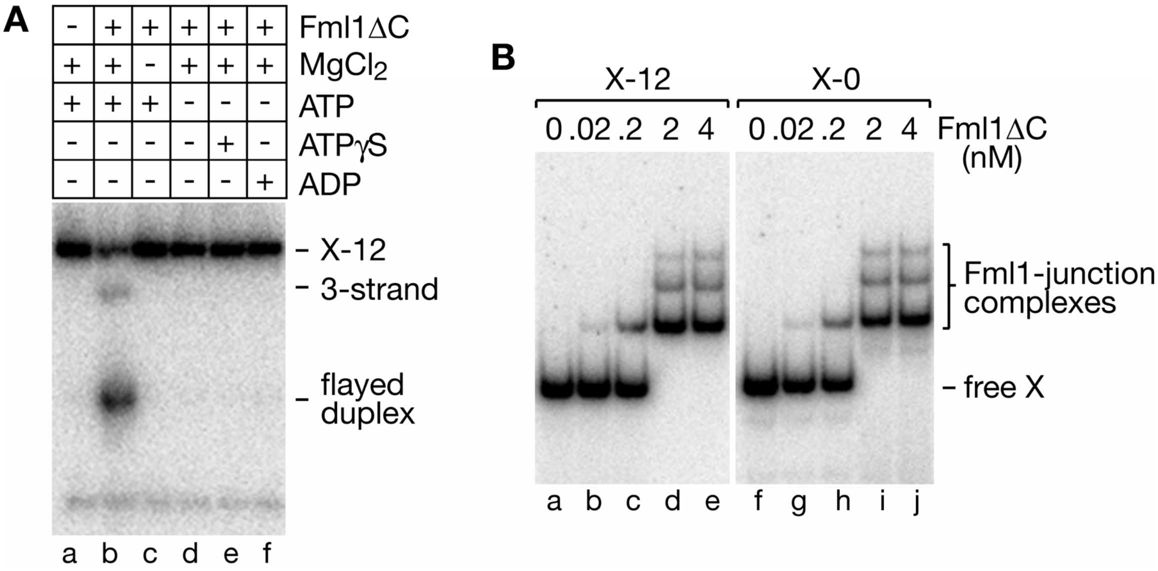

Fig. S5. Binding and dissociation of X-junctions by Fml1ΔC.
(A) Reaction requirements for dissociation of X-12 by Fml1ΔC (2 nM). ATP was replaced with 5 mM ATPγS or ADP as indicated.
(B) EMSA showing binding of Fml1ΔC to X-12 and X-0.

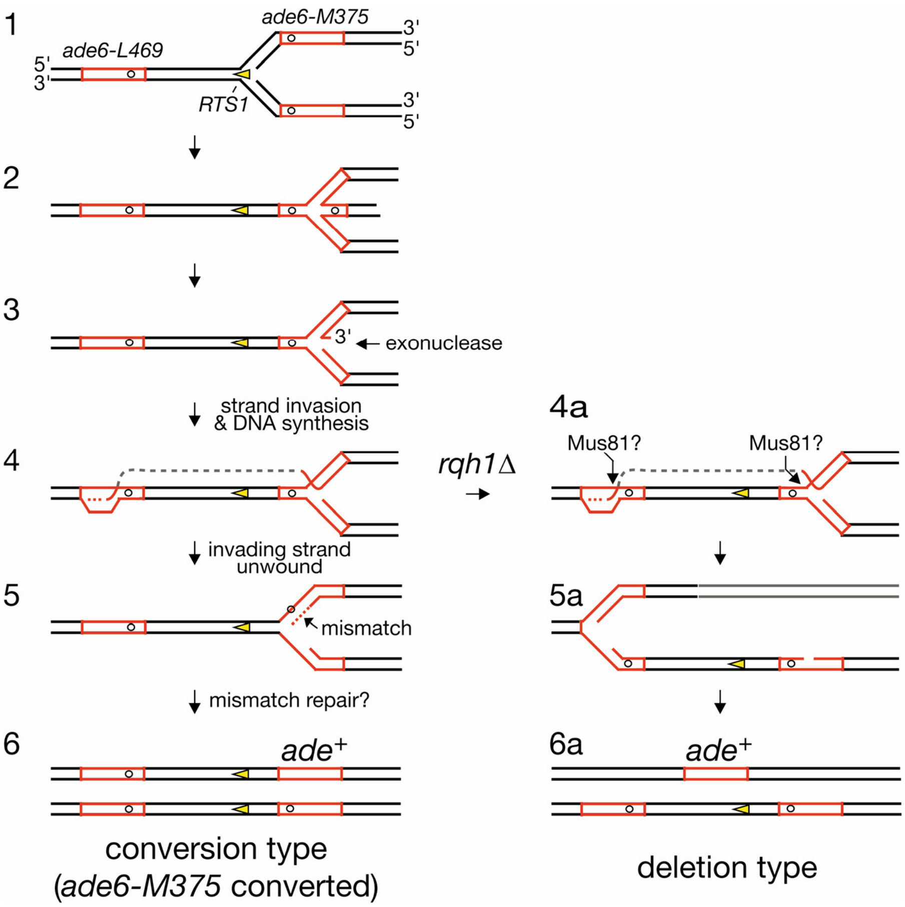

Fig. S6. Hypothetical model for replication fork block-induced recombinant formation in the presence and absence of Rqh1. The circles indicate the point mutations in *ade6-L469* and *ade6-M375*, and the triangle indicates the *RTS1* RFB. This figure is adapted from (Ahn et al., 2005).

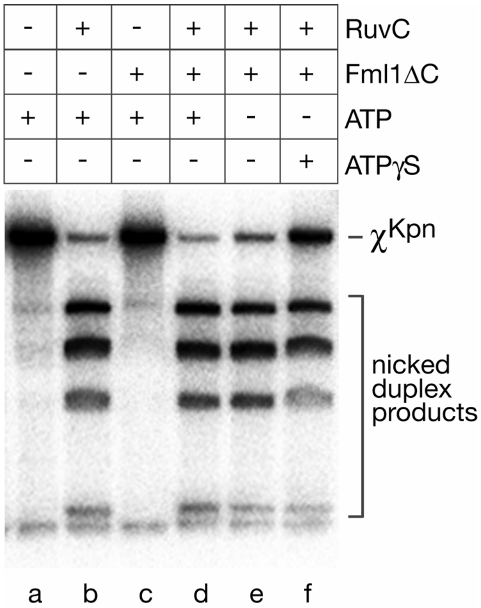

Fig. S7. Cleavage of χ^{Kpn} by RuvC under different reaction conditions as indicated.

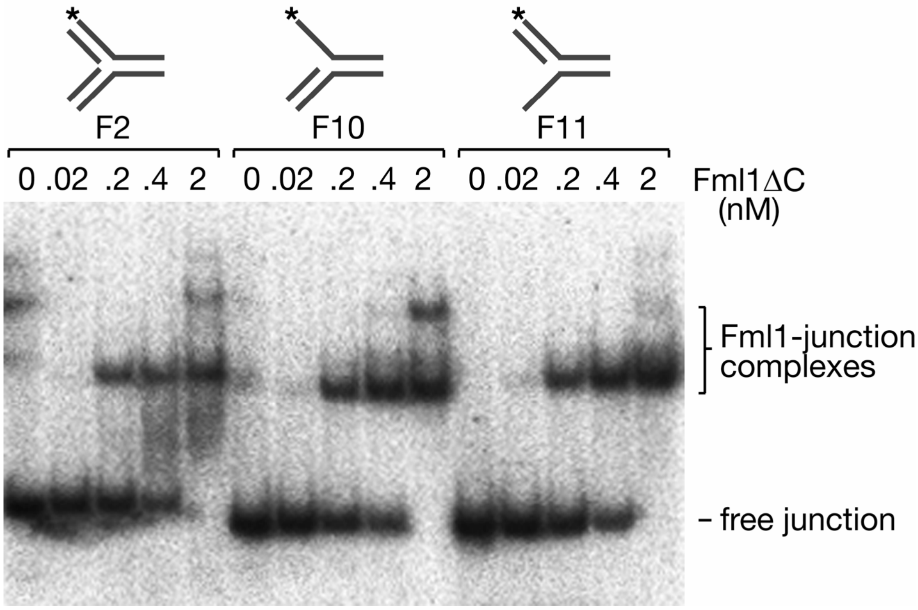

Fig. S8. EMSA showing binding of fork substrates by Fml1ΔC. Reactions (20 µl) contain DNA substrate (0.5 nM) and protein as indicated. Asterisks on the fork schematics indicate the ^{32}P label at the 5' DNA end.

Table S1: Direct repeat recombinant frequencies

Genotype	*RTS1* orientation	Number of colonies analysed[a]	Frequency of conversion-types (x 10^{-4})[b]	*P* value[c]	Frequency of deletion-types (x 10^{-4})[b]	*P* value[d]
wild-type	1	64	1.42 (+/- 0.38)	NA	3.05 (+/- 0.66)	NA
*fml1*Δ	1	55	0.53 (+/- 0.32)	<0.001	2.83 (+/- 1.20)	0.23
*rad51*Δ	1	27	0.07 (+/- 0.11)	<0.001	16.45 (+/- 8.99)	<0.001
*fml1*Δ *rad51*Δ	1	17	0.08 (+/- 0.10)	<0.001	10.85 (+/- 2.95)	<0.001
*rqh1*Δ	1	25	2.95 (+/- 1.12)	<0.001	14.08 (+/- 3.58)	<0.001
*rqh1*Δ *rad51*Δ	1	18	0.14 (+/- 0.28)	<0.001	13.09 (+/- 4.32)	<0.001
*rqh1*Δ *fml1*Δ	1	15	1.80 (+/- 0.72)[e]	0.07	8.64 (+/- 1.96)[e]	<0.001
wild-type	2	63	145.2 (+/- 78.6)	NA	106.1 (+/- 38.3)	NA
*fml1*Δ	2	62	17.1 (+/- 7.7)	<0.001	151.5 (+/- 74.7)	<0.01
*rad51*Δ	2	30	1.82 (+/- 1.27)	<0.001	56.0 (+/- 20.3)	<0.001
*fml1*Δ *rad51*Δ	2	15	2.50 (+/- 1.10)	<0.001	186.6 (+/- 71.8)[f]	0.001
*rqh1*Δ	2	24	394.8 (+/- 200)	<0.001	2660 (+/- 1225)	<0.001
*rqh1*Δ *rad51*Δ	2	19	6.10 (+/- 2.29)	<0.001	76.7 (+/- 24.7)	<0.01
*rqh1*Δ *fml1*Δ	2	15	83.9 (+/- 27.9)[e]	<0.001	807.1 (+/- 314.5)[e]	<0.001

[a] At least 15 colonies from at least three independent experiments were analysed to derive the mean recombinant frequencies shown in this table.
[b] The values in parentheses are the standard deviations about the mean.
[c] *P* value for the comparison of wild-type versus mutant conversion-type frequencies.
[d] *P* value for the comparison of wild-type versus mutant deletion-type frequencies.
[e] Indicated value significantly (*P* <0.001) less than the equivalent *rqh1*Δ single mutant value.
[f] Indicated value significantly (*P* <0.001) greater than the equivalent *rad51*Δ single mutant value.

Table S2: List of *S. pombe* strains used in this study

Strain	Mating type	Genotype	Source
MCW42	h^+	*ura4-D18 his3-D1 leu1-32*	Lab strain
MCW1193	h^+	*ura4-D18 his3-D1 leu1-32 arg3-D4 ade6-M26*	Osman *et al*. 2003
MCW1221	h^+	*ura4-D18 his3-D1 leu1-32 arg3-D4*	Lab strain
MCW1230	h^+	*rhp54Δ::ura4*$^+$ *ura4-D18 his3-D1 leu1-32 arg3-D4*	Lab strain
MCW1231	h^+	*rhp55Δ::arg3*$^+$ *ura4-D18 his3-D1 leu1-32 arg3-D4*	Lab strain
MCW1232	h^+	*rhp55Δ::arg3*$^+$ *rhp54Δ::ura4*$^+$ *ura4-D18 his3-D1 leu1-32 arg3-D4*	This study
MCW1238	h^+	*mus81Δ::kanMX6 ura4-D18 his3-D1 leu1-32 arg3-D4*	Lab strain
MCW1262	h^-	*ura4-D18 his3-D1 leu1-32 arg3-D4 ade6-M375 int::pUC8/his3*$^+$*/RTS1 site A orientation 1/ade6-L469*	Ahn *et al.* 2005
MCW1433	h^-	*ura4-D18 his3-D1 leu1-32 arg3-D4 ade6-M375 int::pUC8/his3*$^+$*/RTS1 site A orientation 2/ade6-L469*	Ahn *et al.* 2005
MCW1443	h^-	*rqh1Δ::kanMX6 ura4-D18 his3-D1 leu1-32 arg3-D4 ade6-M375 int::pUC8/his3*$^+$*/RTS1 site A orientation 1/ade6-L469*	Ahn *et al.* 2005
MCW1447	h^-	*rqh1Δ::kanMX6 ura4-D18 his3-D1 leu1-32 arg3-D4 ade6-M375 int::pUC8/his3*$^+$*/RTS1 site A orientation 2/ade6-L469*	Ahn *et al.* 2005
MCW1691	h^+	*rad51Δ::arg3*$^+$ *ura4-D18 his3-D1 leu1-32 arg3-D4 ade6-M375 int::pUC8/his3*$^+$*/RTS1 site A orientation 1/ade6-L469*	Ahn *et al.* 2005
MCW1692	h^+	*rad51Δ::arg3*$^+$ *ura4-D18 his3-D1 leu1-32 arg3-D4 ade6-M375 int::pUC8/his3*$^+$*/RTS1 site A orientation 2/ade6-L469*	Ahn *et al.* 2005
MCW1712	h^+	*smc6-X-13myc-kanMX6 ura4-D18 his3-D1 leu1-32 arg3-D4*	This study
MCW1723	h^+	*rad51Δ::arg3*$^+$ *smc6-X ura4-D18 his3-D1 leu1-32 arg3-D4*	This study
MCW1818	h^+	*rqh1Δ::kanMX6 ura4-D18 his3-D1 leu1-32 arg3-D4 ade6-M26*	This study
MCW2078	h^-	*fml2Δ::kanMX6 ura4-D18 his3-D1 leu1-32 arg3-D4*	This study
MCW2080	h^+	*fml1Δ::natMX4 ura4-D18 his3-D1 leu1-32 arg3-D4*	This study
MCW2082	h^+	*fml1Δ::natMX4 fml2Δ::kanMX6 ura4-D18 his3-D1 leu1-32 arg3-D4*	This study
MCW2096	h^+	*fml1Δ::natMX4 ura4-D18 his3-D1 leu1-32 arg3-D4 ade6-M26*	This study
MCW2130	h^+	*rad51Δ::arg3*$^+$ *rqh1Δ::kanMX6 ura4-D18 his3-D1 leu1-32 arg3-D4 ade6-M375 int::pUC8/his3*$^+$*/RTS1 site A orientation 2/ade6-L469*	This study
MCW2132	h^+	*rad51Δ::arg3*$^+$ *rqh1Δ::kanMX6 ura4-D18 his3-D1 leu1-32 arg3-D4 ade6-M375 int::pUC8/his3*$^+$*/RTS1 site A orientation 1/ade6-L469*	This study
MCW2264	h^+	*mus81Δ::arg3*$^+$ *fml1Δ::natMX4 ura4-D18 his3-D1 leu1-32 arg3-D4 ade6-M26*	This study
MCW2428	h^+	*mus81Δ::arg3*$^+$ *fml1Δ::natMX4 ura4-D18 his3-D1 leu1-32 arg3-D4*	This study
MCW2487	h^+	*rqh1Δ::his3*$^+$ *fml1Δ::natMX4 ura4-D18 his3-D1 leu1-32 arg3-D4 ade6-M26*	This study
MCW2498	h^+	*rad51Δ::arg3*$^+$ *fml1Δ::natMX4 ura4-D18 his3-D1 leu1-32*	This study

		arg3-D4 ade6-M26	
MCW2550	h^+	*srs2Δ::kanMX6 fml1Δ::natMX4 ura4-D18 his3-D1 leu1-32 arg3-D4 ade6-M26*	This study
MCW3055	h^+	*fml2Δ::kanMX6 ura4-D18 his3-D1 leu1-32 arg3-D4 ade6-M375 int::pUC8/his3$^+$/RTS1 site A orientation 1/ade6-L469*	This study
MCW3057	h^+	*fml2Δ::kanMX6 ura4-D18 his3-D1 leu1-32 arg3-D4 ade6-M375 int::pUC8/his3$^+$/RTS1 site A orientation 2/ade6-L469*	This study
MCW3059	h^+	*fml1Δ::natMX4 ura4-D18 his3-D1 leu1-32 arg3-D4 ade6-M375 int::pUC8/his3$^+$/RTS1 site A orientation 1/ade6-L469*	This study
MCW3061	h^+	*fml1Δ::natMX4 ura4-D18 his3-D1 leu1-32 arg3-D4 ade6-M375 int::pUC8/his3$^+$/RTS1 site A orientation 2/ade6-L469*	This study
MCW3063	h^+	*fml1Δ::natMX4 fml2Δ::kanMX6 ura4-D18 his3-D1 leu1-32 arg3-D4 ade6-M375 int::pUC8/his3$^+$/RTS1 site A orientation 1/ade6-L469*	This study
MCW3065	h^+	*fml1Δ::natMX4 fml2Δ::kanMX6 ura4-D18 his3-D1 leu1-32 arg3-D4 ade6-M375 int::pUC8/his3$^+$/RTS1 site A orientation 2/ade6-L469*	This study
MCW3444	h^+	*fml1Δ::natMX4 rqh1Δ::ura4$^+$ ura4-D18 his3-D1 leu1-32 arg3-D4 ade6-M375 int::pUC8/his3$^+$/RTS1 site A orientation 1/ade6-L469*	This study
MCW3456	h^+	*fml1Δ::natMX4 rqh1Δ::ura4$^+$ ura4-D18 his3-D1 leu1-32 arg3-D4 ade6-M375 int::pUC8/his3$^+$/RTS1 site A orientation 2/ade6-L469*	This study
MCW3671	h^+	*fml1Δ::natMX4 rhp54Δ::ura4$^+$ ura4-D18 his3-D1 leu1-32 arg3-D4*	This study
MCW3701	h^+	*fml1Δ::natMX4 smc6-X-13myc-kanMX6 ura4-D18 his3-D1 leu1-32 arg3-D4*	This study
MCW3770	h^+	*rqh1Δ::kanMX6 fml1Δ::natMX4 ura4-D18 his3-D1 leu1-32 arg3-D4 ade6-M26*	This study
MCW3781	h^+	*rad8Δ::ura4$^+$ ura4-D18 his3-D1 leu1-32 arg3-D4*	This study
MCW3790	h^+	*fml1Δ::natMX4 rad51Δ::arg3$^+$ ura4-D18 his3-D1 leu1-32 arg3-D4 ade6-M375 int::pUC8/his3$^+$/RTS1 site A orientation 1/ade6-L469*	This study
MCW3794	h^+	*fml1Δ::natMX4 rad51Δ::arg3$^+$ ura4-D18 his3-D1 leu1-32 arg3-D4 ade6-M375 int::pUC8/his3$^+$/RTS1 site A orientation 2/ade6-L469*	This study
MCW3811	h^+	*srs2Δ::kanMX6 ura4-D18 his3-D1 leu1-32 arg3-D4 ade6-M26*	This study
MCW3816	h^+	*fml1Δ::natMX4 rad8Δ::ura4$^+$ ura4-D18 leu1-32*	This study
FO1192	h^+	*mus81Δ::kanMX6 ura4-D18 his3-D1 leu1-32 arg3-D4 ade6-M26*	This study

Table S3: DNA substrates

oligo 1
5'-GACGCTGCCGAATTCTGGCTTGCTAGGACATCTTTGCCCACGTTGACCC-3'
oligo 2
5'-TGGGTCAACGTGGGCAAAGATGTCCTAGCAATGTAATCGTCTATGACGTT-3'
oligo 3
5'-CAACGTCATAGACGATTACATTGCTAGGACATGCTGTCTAGAGACTATCGA-3'
oligo 4
5'-ATCGATAGTCTCTAGACAGCATGTCCTAGCAAGCCAGAATTCGGCAGCGT-3'
oligo 5
5'-TGCCGAATTCTACCAGTGCCAGTGATGGACATCTTTGCCCACGTTGACCC-3'

oligo 6
5'-GTCGGATCCTCTAGACAGCTCCATGATCACTGGCACTGGTAGAATTCGGC-3'

oligo 7
5'-CAACGTCATAGACGATTACATTGCTACATGGAGCTGTCTAGAGGATCCGA-3'
oligo 8
5'-CAACGTCATAGACGATTACATTGCTAATCACTGGCACTGGTAGAATTCGGC-3'
oligo 10
5'-GGACATCTTTGCCCACGTTGACCC-3'

oligo 15
5'-TGCCGAATTCTACCAGTGCCAGTGAT-3'

oligo 16
5'-GACGCTGCCGAATTCTACCAGTGCCTTGCTAGGACATCTTTGCCCACCTGCAGGTTCACCC-3'

oligo 17
5'-GGGTGAACCTGCAGGTGGGCGGCTGCTCATCGTAGGTTAGTTGGTAGAATTCGGCAGCGTC-3
oligo 18
5'-AAAGATGTCCTAGCAAGGCAC-3'
oligo 19
5'-TAAGAGCAAGATGTTCTATAAAAGATGTCCTAGCAAGGCAC-3'
oligo 22
5'-TATAGAACATCTTGCTCTTA-3'

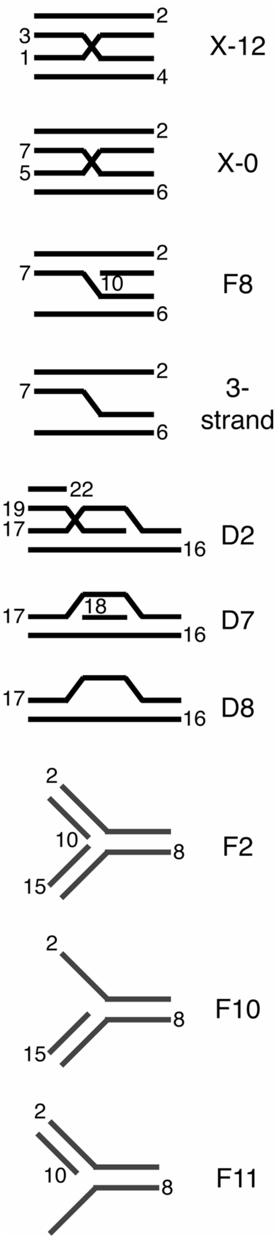

Each DNA substrate is made from the oligonucleotides indicated by the number on each representative schematic. The number is positioned at the 5'-end of its respective oligonucleotide. Note that the 3-strand junction is called F9 in the paper.
